# Pantothenate Kinase-Associated Neurodegeneration (PKAN) With Concomitant Blepharospasm: Unveiling a Clinical Enigma

**DOI:** 10.7759/cureus.46665

**Published:** 2023-10-08

**Authors:** Venkat Reddy, Keyur Saboo, Kavyanjali Reddy, Sunil Kumar, Sourya Acharya

**Affiliations:** 1 General Medicine, Jawaharlal Nehru Medical College, Datta Meghe Institute of Higher Education and Research, Wardha, IND; 2 Medicine, Jawaharlal Nehru Medical College, Datta Meghe Institute of Higher Education and Research, Wardha, IND; 3 Surgery, Jawaharlal Nehru Medical College, Datta Meghe Institute of Higher Education and Research, Wardha, IND

**Keywords:** case report, vitamin b5, young male, diagnosis of pkan, blepharospasm

## Abstract

Pantothenate kinase-associated neurodegeneration (PKAN) is a rare and complex neurodegenerative disorder. It occurs due to mutations in the sequencing of the PANK2 gene. Here, we describe the case of a 22-year-old male patient who presented with severe blepharospasm; he had abnormal facial distortions, shaky limbs, rigid muscles, and a slow pace of movement, making a diagnosis tricky. Accumulation of iron in excessive amounts in the basal ganglia, a part of the brain that governs movement, is linked to PKAN. In this case, the "eye of the tiger" indication, a distinctive pattern only seen by MRI, supported PKAN. The anticholinergic medications helped him alleviate his symptoms to some extent, but he still had some degree of impairment. This instance emphasizes the mysterious character of PKAN and the significance of keeping an eye out for unusual symptoms in neurodegenerative conditions. This case report emphasizes the significance of recognizing unexpected effects that brain disorders can have on people's lives and calls for increased clinician awareness and understanding.

## Introduction

In 1922, Hallervorden and Spatz first described pantothenate kinase-associated neurodegeneration (PKAN), a rare neurogenerative disorder, marking a crucial turning point in medical history. This illness, which has an indistinct childhood onset, embarks on an aggressive path of advancement and presents a series of confusing symptoms that when combined provide a complex clinical picture [1]. Among these symptoms, manifestations like choreoathetosis (involuntary, writhing movements) and hyperkinesia (excessive, uncontrolled movements) intricately add to the clinical tapestry. Other manifestations include bradykinesia (slow movement), rigidity (stiff muscles), spasticity (muscle tightness), and dystonia (abnormal body movements) [2].

PANK2 gene mutations, which have an amazingly narrow genetic influence and only affect one out of three per million people, are linked to the development of this rare condition [3]. The neurodegeneration with brain iron accumulation (NBIA) spectrum encompasses various forms of neurodegenerative diseases, each with a unique story. Pantothenate kinase-associated neurodegeneration (NBIA1), also known as PKAN, occurs as a result of a complicated series of mutations in the sequencing of the PANK2 gene. Phospholipase A2-associated group VI (PLA2G6) neurodegeneration (NBIA2), or PLAN, carries a distinctive genetic signature in the PLA2G6 gene. Neuroferritinopathy occurs due to mutations in the ferritin light chain gene, or the FTL gene. Aceruloplasminemia is caused due to mutations in the ceruloplasmin gene. In cases with unknown genetic relationships, there is a mystery around occasional NBIA occurrences, whose genetic causes are not yet known.

The aim of this case study is to give a thorough analysis of the treatment received by a PKAN patient, highlighting the challenging diagnostic procedure, clinical symptoms, and treatment options related to pantothenate kinase-associated neurodegeneration.

## Case presentation

A 22-year-old male patient presented to the medicine outpatient department with involuntary tight closure of both eyelids with difficulty in eye opening, for three months (Video 1). Prior to this, he was receiving treatment from a local general practitioner, but since there was no improvement in his condition, he was referred to the tertiary care hospital (Acharya Vinoba Bhave Rural Hospital, Wardha, Maharashtra, India). There was no history of head trauma, no family history of movement disorders, and no history of consanguinity. He denied any history of taking medications like antiemetics (metoclopramide, etc.). He was a nonalcoholic and nonsmoker.

**Video 1:** Video showing the involuntary tight closure of both eyelids with difficulty in eye opening

After around 48 hours of admission, his symptoms worsened, as he started to have tremors in his limbs and unusual facial expressions such as frequent grimacing, tongue protrusion, and lip-smacking. Upon inspection, there was evidence of apraxia of eyelid opening and bilateral eyelid closure. On examination, he also had increased muscle tone suggesting spasticity. His routine laboratory workup showed that he had a significant deficiency of vitamin B5 (Table 1).

**Table 1:** Laboratory investigation profile of the patient

Investigations	Patient	Reference values
Hemoglobin	10.8 g/dl	13-17 g/dl
Total leukocyte count	8700/dl	4000-11000/dl
Platelet count	252,000/dl	150,000-400,000/dl
Serum creatinine	0.6 mg/dl	0.5-1.2 mg/dl
Albumin	4.2 g/dl	3.5-5.0 g/dl
Aspartate aminotransferase	48 U/l	<50 U/l
Alanine aminotransferase	58 U/l	17-59 U/l
Total bilirubin	0.5 mg/dl	0.2-1.3 mg/dl
Ferritin	73.2 ng/ml	17-464 ng/ml
Serum iron	98 ug/dl	49-181 ug/dl
Vitamin B5	8.2 ug/l	23-430 ug/l
Vitamin B12	456 pg/ml	150-950 pg/ml

**Figure 1:**
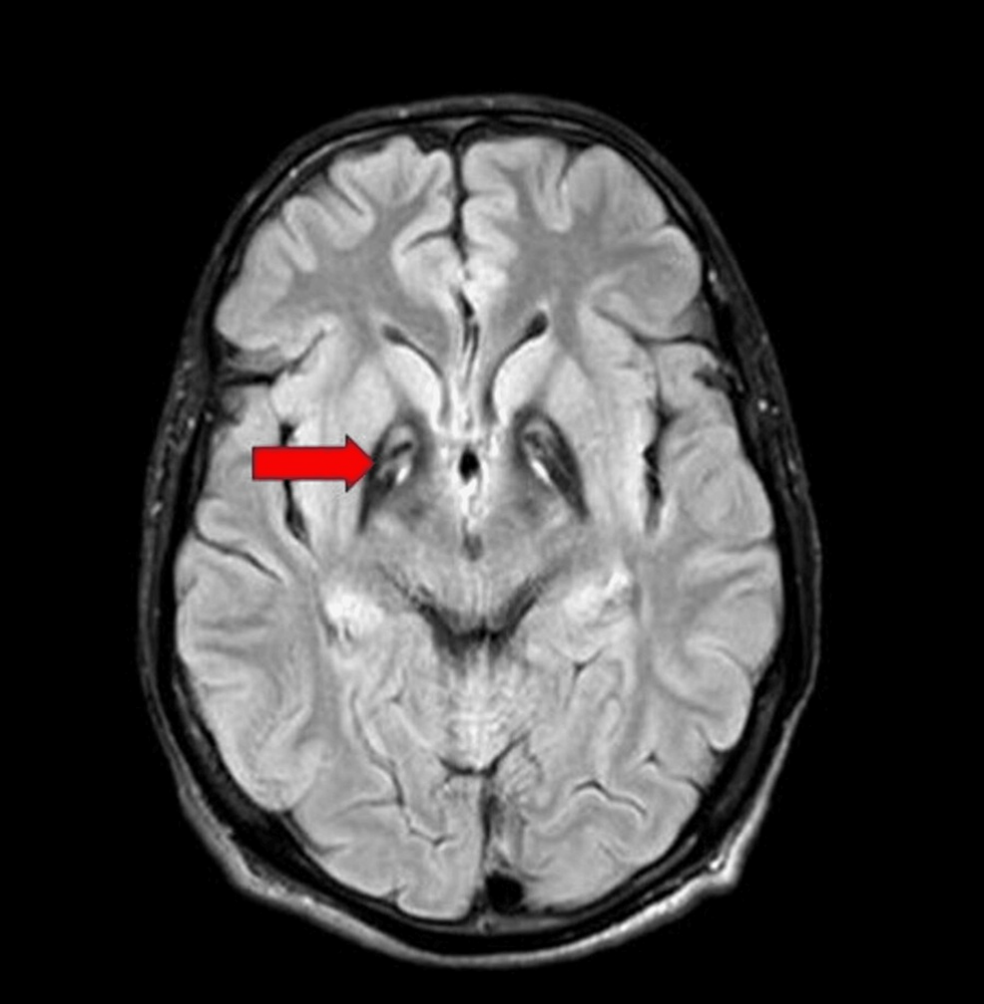
Brain MRI showing globus pallidus having bilateral and symmetrical hypodensities as well as the medial zone of hyperintensity with recognizable "eye of the tiger" sign (red arrow)

In view of these movement disorders, brain MRI was performed, which revealed bilateral and symmetrical hypodensities of the globus pallidus on T2-weighted Images. Surprisingly, this area's medial zone of hyperintensity matched the recognizable "eye of the tiger" sign (Figure 1). His genetic testing identified a well-documented homozygous deleterious mutation (881A>T/p.N294I) and supported the PKAN diagnosis. The patient received injectable vitamin B5 (pantothenic acid). The patient was given trihexyphenidyl 5 mg three times a day and baclofen 10 mg twice daily for spasticity. Symptoms improved after seven days, including reduced tremors, improved eye-opening, and steadier gait (Video 2). Injectable vitamin B5 was given for 10 days, followed by the oral form until discharge after 15 days. Follow-up after one month showed minimal symptoms or signs.

**Video 2:** Video showing improvement in eye opening

## Discussion

Regardless of the accurate form, focal dystonias are a common issue that runs through PKAN's presentation. Children commonly exhibit an unstable walk as part of the classic expression of limb dystonia. Surprisingly, pigmentary retinopathy manifests relatively early in approximately two-thirds of children with classic PKAN. The possibility of blindness from this ocular involvement adds another level of complexity to the clinical environment [4,5].

The diagnosis of PKAN is often made based on a combination of radiological findings and clinical suspicion. Frequently, the clinical characteristics resemble a hint, gradually cultivating a strong suspicion that is finally confirmed with the disclosure of the distinctive brain MRI pattern. The PKAN phenotypic spectrum's landscape has changed as a result of the discovery of the PANK2 gene, opening up a wider range of presenting traits. The time between the onset of early clinical symptoms and an accurate diagnosis for affected people may be significantly reduced with the use of comprehensive clinical whole-exome sequencing in combination with improved accessibility to early neuroimaging via MRI sequences adjusted to iron sensitivity [6,7].

Brain MRI using iron-sensitive MRI sequences such as susceptibility weighted imaging (SWI), gradient echo sequences (GRE), and T2* stands as a leading diagnostic strategy to reveal unique alterations. With this technique, the amazing "eye of the tiger" sign comprising hypointense periphery enclosing a central hyperintense region within the globus pallidus can be seen on T2-weighted imaging [8,9].

Genetic testing is necessary to confirm the diagnosis when PKAN is suspected. It is essential to do a thorough analysis that includes sequencing and deletion/duplication studies of the PANK2 gene, which is crucial in PKAN mutations. Beyond diagnosis, genetic information provides helpful prognostic hints, opens the door to future prenatal or pre-implantation genetic diagnoses, and identifies carriers in the context of the family. Resources for clinical genetic testing are available internationally through regional experts in medical genetics or neurology [10].

A report from Natteru and Huang highlighted the case of a 50-year-old patient who had pantothenate kinase-associated neurodegeneration [11]. The patient had a long history of stuttering speech and was initially misdiagnosed with Parkinson's disease. His treatment included trihexyphenidyl and amantadine, and Botox injections were used to treat his blepharospasm, resulting in significant improvement. Diaz reported a case of a 50-year-old female patient who presented with difficulty swallowing, slurred speech, and uncontrolled eyelid spasms. She was treated with a Botox injection and showed considerable improvement [12].

According to clinical characteristics, our patient had the atypical PKAN variety; this was distinguished by its commencement in the second or third decade of life, progressing gradually over the course of the disease.

There is no effective treatment for PKAN patients, and the many medications on the market have no impact on the disease's progression. Patients with dystonia may experience substantial disability as the condition worsens over time and spreads to various body regions. Anticholinergics, botulinum toxin, benzodiazepines, clonidine, gabapentin, pregabalin, tetrabenazine, and other anti-spasticity medications are frequently used to treat it, alone or in combination. By raising coenzyme A levels and reversing mitochondrial dysfunction, vitamin B5 (pantothenate) may be able to help PKAN patients. Surgical procedures, such as deep brain stimulation, are currently used to treat PKAN disorder. Deep brain stimulation can have positive effects soon after the surgery, but because the disease is progressive, symptoms may return later and the condition may worsen. Currently, deferiprone, deferoxamine, and deferasirox are used as iron chelators. Reportedly, deferiprone can get beyond the blood-brain barrier and has iron-relocating and redistributing abilities that can prevent cerebral iron accumulation. In a small, unblinded pilot trial including four patients with genetically confirmed PKAN, and two with parkinsonism and focal dystonia, but inconclusive genetic tests, the efficacy of deferiprone was evaluated. Surprisingly, MRI revealed decreased iron accumulation in the globus pallidus of two patients (one with PKAN) and mild-to-moderate motor improvement in three patients (two with pantothenate kinase-associated neurodegeneration) [13]. However, significant clinical improvement or an improvement in quality of life eluded researchers. This predictive environment sets the direction for the ongoing deferiprone research.

## Conclusions

This case report reveals the difficult diagnostic journey of a 22-year-old male patient who was eventually diagnosed with the mysterious pantothenate kinase-associated neurodegeneration condition, highlighting the complex clinical range of PKAN. Advanced imaging and genetic testing enable rapid and precise diagnosis, which not only provides clinical relief but also helps with prognosis and genetic counseling. Efficacy of possible treatments including deep brain stimulation and botulinum toxin A injections is also discussed. This case emphasizes the value of interdisciplinary cooperation in addressing the complex problems posed by PKAN and related uncommon neurodegenerative disorders and calls for increased clinician awareness.
